# The Combined Effects of Toxic *Microcystis aeruginosa* and Thermal Stress on the Edible Clam (*Corbicula fluminea*): Insights into Oxidative Stress Responses and Molecular Networks

**DOI:** 10.3390/antiox12111901

**Published:** 2023-10-24

**Authors:** Jingxiao Zhang, Ning Wang, Zehao Zhang, Yunni Gao, Jing Dong, Xiaofei Gao, Huatao Yuan, Xuejun Li

**Affiliations:** 1 College of Fisheries, Henan Normal University, Xinxiang 453007, China; 2 Observation and Research Station on Water Ecosystem in Danjiangkou Reservoir of Henan Province, Nanyang 473000, China

**Keywords:** microcystin, thermal stress, oxidative stress responses, transcriptome analysis

## Abstract

Cyanobacterial blooms (CYBs) have become a global environmental issue, posing risks to edible bivalves. Toxic cyanobacteria and thermal stress represent the two key co-occurring stressors to bivalves experiencing CYBs. To investigate the combined effects of these stressors on the edible bivalve *Corbicula fluminea*, the responses to oxidative stress and the molecular mechanisms of physiological adaptations in *C*. *fluminea* were examined under co-exposure to toxic *Microcystis aeruginosa* and thermal stress. The activity of antioxidant enzymes, including GST, SOD, CAT, GPx and GR, was significantly influenced by the interaction between temperature and *M. aeruginosa* (*p* < 0.05). A positive correlation was observed between toxic *M. aeruginosa* exposure and elevated SOD and GPx activities at 30 °C, demonstrating that SOD and GPx may help *C. fluminea* defend effectively against MCs under thermal stress. Furthermore, significant interactive effects between toxic *M. aeruginosa* and temperature were also observed in ROS and MDA (*p* < 0.05). The results of the PCA and IBR index also evidenced the apparent influence of toxic *M. aeruginosa* and thermal stress on oxidative stress responses of *C. fluminea*. The eggNOG and GO annotations confirmed that a substantial portion of differentially expressed genes (DEGs) exhibited associations with responses to oxidative stress and transporter activity. Additionally, KEGG analysis revealed that abundant DEGs were involved in pathways related to inflammatory responses, immune functions and metabolic functions. These findings improve our understanding of the mechanism of the physiological adaptation in bivalves in response to cyanotoxins under thermal conditions, potentially enabling the evaluation of the viability of using bivalves as a bioremediation tool to manage CYBs in eutrophic waters.

## 1. Introduction

Due to elevated eutrophication from intensified human activities (e.g., agricultural activities, urbanization) and global warming, cyanobacterial blooms (CYBs) have been increasing in frequency, intensity and duration worldwide [1]. One of the primary risks associated with CYBs is the synthesis of various cyanotoxins. Microcystins (MCs), known as one of the most toxic cyanotoxins produced by *Microcystis* strains, have been the subject of numerous studies and have attracted extensive attention due to their high toxicity and widespread occurrence [2]. MCs are generally recognized as tumor promoters, with chronic exposure to MCs associated with the appearance of liver cancer, hence posing a serious risk to human health [3]. As typical hepatotoxins, MCs can directly affect the liver or hepatopancreas of aquatic organisms, causing oxidative stress, liver damage and even reducing growth and reproductive capacity [4].

As global warming increases, many eutrophic waters are experiencing significant fluctuations in temperature. Numerous studies have indicated that elevated temperatures have the potential to provide advantages to toxic bloom-forming cyanobacteria by increasing their growth rate and expanding their ecological niche [5,6,7]. Therefore, the observed rise in temperatures has been associated with an increased proliferation and a prolonged duration of toxic CYBs [8]. For aquatic animals, elevated temperatures could enhance their physiological metabolism’s performance and increase oxygen consumption, thus potentially leading to an increase in reactive oxygen species (ROS) levels and the induction of oxidative stress [9]. Although the individual effects of toxic CYBs and thermal stress on aquatic animals have been well characterized, there is a lack of information on the combined effects of toxic CYBs and elevated temperatures. Previous studies have assessed the toxicity of cyanobacteria at different temperatures in aquatic animals, including zebrafish (*Danio rerio*), mussels (*Hyriopsis cumingii*), freshwater daphnids (*Moina macrocopa*) and rotifers (*Brachionus calyciflorus*), indicating that the combined effects of temperature and cyanotoxins are complicated and species-specific [9,10,11,12,13]. Consequently, a comprehensive understanding of the combined impacts of toxic cyanobacteria and thermal stress on various aquatic organisms will provide a broader perspective on the sustainability of aquatic ecosystems.

Bivalves generally inhabit the benthic zone and play an important role in the trophic structure of freshwater ecosystems, which can effectively facilitate energy flows and nutrient cycling within the ecosystem [14]. Compared to other aquatic organisms, bivalves exhibit more susceptibility to the effects of CYBs due to their ability to filter feed on cyanobacteria as well as their restricted migration capacity [15]. The edible bivalve *Corbicula fluminea*, formerly endemic to Eastern Asia and Southeast Asia, has achieved global distribution as a result of its high fertility and adaptability to various habitats [16]. *C. fluminea* has significant economic value, not only for direct human consumption, but also as a feed option for livestock and poultry, as well as a natural bait for carnivorous fish and waterfowl [17]. As a model bivalve, *C. fluminea* has been widely used for the evaluation of the toxic effects of various pollutants due to its low mobility, ease of cultivation, economic practicality and wide distribution [18,19,20]. Furthermore, *C. fluminea* has also been considered a valuable tool for the restoration of eutrophic lakes where toxic CYBs occur due to their efficient filtration of bloom-forming cyanobacteria and their ability to tolerate cyanotoxins [21,22]. Previous studies have demonstrated that toxic *M. aeruginosa* or thermal stress could affect the growth performance, biochemical parameters and physiological functions of *C. fluminea* [23,24,25]. However, little is known about the combined effects of toxic *M. aeruginosa* and thermal stress on this species. Furthermore, there is still a lack of information on the molecular mechanisms of tolerance to MCs in *C. fluminea* at high temperatures.

Most of the evidence indicates that the adverse effects of MC exposure and thermal stress on aquatic animals could be attributed to ROS production [26]. The presence of an excessive amount of ROS has the potential to cause lipid peroxidation in cell membranes, protein denaturation and DNA damage [9]. Cell antioxidant defense systems, including a series of antioxidant enzymes such as superoxide dismutase (SOD), catalase (CAT), glutathione peroxidase (GPx) and glutathione reductase (GR), possess the ability to maintain redox equilibrium and protect cells against oxidative damage [25]. Oxidative stress arises as a result of an imbalance between ROS-induced oxidative effects and ROS elimination mediated by antioxidant defense systems. Therefore, these biochemical parameters are commonly employed as biomarkers in the evaluation of the biological toxicity responses of MCs in bivalves. Additionally, the deployment of high-throughput RNA sequencing technology (RNA-seq) has enabled the comprehensive analysis of transcriptomes, resulting in their increasing use in the study of the molecular regulatory mechanisms of aquatic animals in response to MC exposure [27,28].

This study investigated the combined effects of toxic *M. aeruginosa* and thermal stress on oxidative stress responses, including GST, SOD, CAT, GPx and GR activity, ROS level and malondialdehyde (MDA) content of the edible clam *C. fluminea*. Moreover, a principal component analysis (PCA) and an integrated biomarker response (IBR) analysis were performed to highlight the integrated variations in biochemical parameters between different treatment groups (three different concentrations of toxic *M. aeruginosa* × three temperature gradients). Furthermore, the RNA-seq was conducted in the hepatopancreas to determine the molecular response in *C. fluminea* exposed to toxic *M. aeruginosa* under thermal stress. The present study could contribute to further understanding of the physiological adaptation mechanisms of edible bivalves to co-exposure to toxic cyanobacteria and thermal stress, potentially enabling the evaluation of the feasibility of bivalves as a bioremediation tool to control CYBs in eutrophic waters.

## 2. Materials and Methods

### 2.1. Experimental Clam Acclimation and Algae Culture

Healthy *C. fluminea* was obtained from Hongze Lake (China, 118.8° E, 33.3° N). Clams (length 2.16 ± 0.23 cm) were transported to the laboratory and acclimated for at least 14 days under controlled conditions, with a water temperature of 20 ± 1 °C, dissolved oxygen content greater than 6.0 mg/L, a pH of 7.4 ± 0.3 and a 12 h light–dark photoperiod. The clams were fed *Chlorella vulgaris* (1 × 10^5^ cells/mL) twice daily during the acclimatization period. The algae species, namely toxic *M. aeruginosa* (FACHB-905) and *C*. *vulgaris* (FACHB-8), were obtained from the Institute of Hydrobiology, CAS (Wuhan, China). The microalga was cultured in BG11 medium and harvested in stationary phase with a density of 0.5–1 × 10^7^ cells/mL.

### 2.2. Experimental Design

The acclimated clams were exposed to nine treatments (3 × 3 factorial design), including three temperature levels (20, 25 and 30 °C) and three toxic *M. aeruginosa* concentrations (0, 1 and 10 × 10^5^ cells/mL). The treatment groups without *M. aeruginosa* exposure (0 cells/mL) were fed with *C. vulgaris* (1 × 10^5^ cells/mL) instead. Forty clams were randomly assigned to each treatment group and cultured in 20 L tanks. Each treatment was performed in triplicate. During the experiment, full water renewal was performed once daily with the addition of microalgae.

Five clams from each tank were randomly collected for biochemical parameter determinations on days 1, 3, 5 and 7. After 7 days of exposure, ten clams from the control group (0 cells/mL toxic *M. aeruginosa* × 20 °C) and the treatment group (10 × 10^5^ cells/mL *M. aeruginosa* × 30 °C) were collected for transcriptome analysis.

### 2.3. Determination of Biochemical Parameters

The hepatopancreas of five sampled clams, weighing about 0.2 g, were homogenized via sonication in the ice bath. The supernatant was produced via centrifugation (2500 rpm for 10 min at 4 °C) and then used for the determination of biochemical parameters. Biochemical parameters were evaluated using assay kits (Nanjing Jiancheng Institute of Bioengineering, Nanjing, China) as per the manufacturer’s protocols. In summary, the determination of GST activity was conducted using the colorimetric approach, employing the GST Assay Kit. The measurement of SOD activity was conducted via the SOD Assay Kit using the WST-1 method. The CAT Assay Kit was utilized to evaluate the CAT activity by measuring the rate of the hydrogen peroxide (H_2_O_2_) decomposition reaction. The activity of GPx was evaluated using the GSH-PX Assay Kit, which quantifies the rate of the catalytic reaction involving glutathione. The assessment of GR activity was performed by quantifying the reduction in absorbance at 340 nm, which is indicative of NADPH consumption. Furthermore, the determination of MDA content was performed via the thiobarbituric acid method, employing the MDA Assay Kit. The ROS Assay Kit was employed to quantify ROS levels using the DCFH-DA probe method. The protein content was evaluated via the Protein Assay Kit, applying the Coomassie Brilliant Blue method.

### 2.4. The IBR Index Analysis

The integrated biomarker response index was calculated from the biochemical variables investigated. Calculation of IBR values was carried out using the formulae proposed by Sanchez et al. [29]:

(1) $$Y_{i}=log\frac{X_{i}}{X_{0}}$$

(2) $$Z_{i}=\frac{Y_{i}-\mu }{\sigma }$$

(3) $${A=Z}_{i}-Z_{0}$$

(4) $$IBR=\sum \left|A\right|$$

The variables $X_{i}$ and $X_{0}$ represent the individual biomarker data and the mean reference data, respectively. $Y_{i}$ denotes the logistic transformation of the individual biomarker’s data. μ and σ denote the average and standard deviation of $Y_{i}$. Furthermore, $Z_{i}$ represents the average of the standardized biomarker response, while $Z_{0}$ represents the average of the reference biomarker data. Finally, A denotes the deviation in reference values for each biomarker.

### 2.5. Transcriptome Analysis

The hepatopancreas of sampled clams were dissected for RNA extraction. Libraries were prepared using the VAHTS^®^ Universal V6 RNA-Seq Library Prep Kit and then subjected to sequencing on the Illumina NovaSeq 6000 platform. The differential expression analysis was conducted using the DESeq2 package developed by Anders and Huber [30]. To identify genes that exhibit differential expression, a fold change (FC) > 2 and a false discovery rate (FDR) < 0.05 were used. Functional annotations and enrichment analyses of differentially expressed genes (DEGs) were conducted using the evolutionary genealogy of genes: Non-supervised Orthologous Groups (eggNOG), the Gene Ontology (GO) and the Kyoto Encyclopaedia of Genes and Genomes (KEGG) databases.

### 2.6. Statistical Analyses

Statistical analysis was performed using SPSS 25 software (IBM, New York, NY, USA). Data for the biochemical parameters determined were expressed as mean ± SD. Statistical tests of normality (Shapiro–Wilk’s test, 1% risk) and equal variance (Levene’s test, 5% risk) were employed for the results prior to analysis. The pair correlation coefficients across biochemical parameters calculated by Pearson correlation were shown in Figure S1. A three-way analysis of variance (ANOVA) was used to analyze the effects of temperature, toxic *M. aeruginosa* and time course on variations of biochemical parameters, followed by Tukey’s test to identify pairwise differences. The level of significance was defined as *p* < 0.05. Principal component analysis of these parameters was performed using GraphPad Prism 9.3 software (GraphPad Software, Boston, MA, USA).

## 3. Results and Discussion

### 3.1. Oxidative Stress Induced by Thermal Stress and Toxic M. aeruginosa

#### 3.1.1. Activation of Detoxification

**Figure 1:**
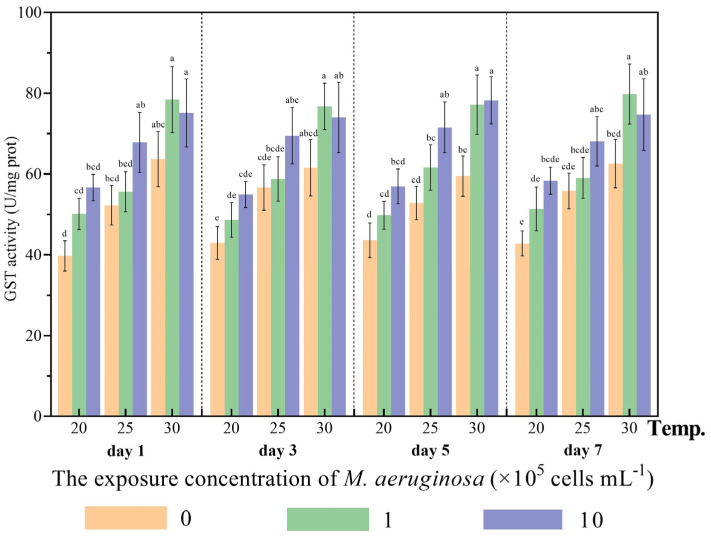
The activities of the glutathione S−transferase (GST) of *C*. *fluminea* exposed to nine combinations of toxic *M. aeruginosa* (0, 1 and 10 × 10^5^ cells/mL) and temperatures (20, 25 and 30 °C) at days 1, 3, 5 and 7. Different letters indicate significant differences among treatment groups at each sampling time (Tukey HSD test, *p* < 0.05). Error bars indicate means ± SD (*n* = 3).

GST is responsible for facilitating the conjugation of electrophilic substrates with glutathione (GSH), thus serving a crucial function in detoxification and defending aquatic organisms from the harmful effects of oxidative metabolic by-products [31]. At low and medium temperatures (20 and 25 °C), the treated clams exhibited a gradual increase in GST activity as the levels of toxic *M. aeruginosa* increased, regardless of the duration of exposure (Figure 1). No statistically significant differences were observed between different diet treatments at 20 °C (*p* > 0.05). However, under high temperature conditions (30 °C), GST activity in the treatment groups that were fed low concentrations (1 × 10^5^ cells/mL) of toxic *M. aeruginosa* was significantly increased compared to those fed *C. vulgaris* at sampling times on days 5 and 7, indicating that *C. fluminea* could exhibit a higher metabolic rate towards low concentrations of *M. aeruginosa* at high temperatures (30 °C). The three-way ANOVA results showed that temperature and *M. aeruginosa* significantly affected the GST activity of *C. fluminea* (*p* < 0.05). Furthermore, significant interactions between temperature and *M. aeruginosa* for GST activity were observed in treated clams (*p* < 0.05). This may indicate that GST-mediated metabolism enables *C. fluminea* to endure cyanobacterial blooms but may be affected by thermal stress. In *H. cumingii*, similar elevated GST activity was observed and GST activity was obviously affected by temperature and toxin concentration [9]. The comparable phenomenon of increased GST activity under MC exposure can also be observed in marine bivalves, such as *M. edulis* and *C. gigas* [32]. Burmester et al. [33] found that the GST of invasive bivalves could respond more strongly to cyanotoxins than that of indigenous species. Consequently, the physiological adaptations to CYBs observed in *C. fluminea* could be attributed to the increased activities of biotransformation enzymes in response to cyanotoxins under thermal stress.

#### 3.1.2. Influence on Antioxidant Defense Systems

**Figure 2:**
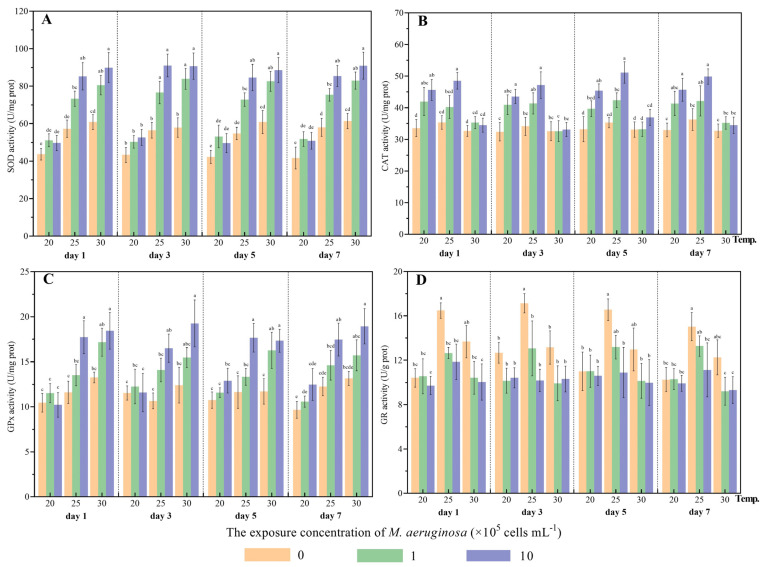
The activities of antioxidant enzymes of *C*. *fluminea* exposed to nine combinations of toxic *M. aeruginosa* (0, 1 and 10 × 10^5^ cells/mL) and temperatures (20, 25 and 30 °C) at days 1, 3, 5 and 7. (**A**) superoxide dismutase (SOD); (**B**) catalase (CAT); (**C**) glutathione peroxidase (GPx); (**D**) glutathione reductase (GR). Different letters indicate significant differences among treatment groups at each sampling time (Tukey HSD test, *p* < 0.05). Error bars indicate means ± SD (*n* = 3).

SOD and CAT generally serve as the primary defense mechanisms in bivalves against oxidative stress induced by exogenous toxic substances [34]. SOD can facilitate the production of H_2_O_2_ through the catalysis of O_2_^−^, which subsequently involves its degradation by CAT into water and O_2_ [35]. Therefore, the transformation of excess ROS (such as O_2_^−^ and H_2_O_2_) into harmless metabolites can be linked to elevated SOD and CAT activities. As the second line of defense against oxidative stress, GPx could facilitate the conversion of reduced GSH to GSH disulfide, thus removing excess H_2_O_2_ [32]. In addition, GR plays a crucial role in catalyzing the reduction of GSH disulfide to GSH [36]. In the present study, no significant differences were observed for SOD activity between different diet treatment groups at 20 °C (Figure 2). However, at conditions of 25 and 30 °C, a significant increase in SOD activity was observed in treated clams fed toxic *M. aeruginosa* (1 and 10 × 10^5^ cells/mL) compared to those fed *C. vulgaris* at each sampling time. On the contrary, compared to the treatment groups fed *C. vulgaris*, CAT activities increased significantly in the treatment groups which were fed high concentrations of *M. aeruginosa* (10 × 10^5^ cells/mL) at 20 and 25 °C, while no significant differences were observed between the different diet treatments at 30 °C (Figure 2). GPx activity exhibited similar fluctuations to SOD activity during the co-exposure period to *M. aeruginosa* and thermal stress (Figure 2). GPx activity gradually increased as the concentration of toxic *M. aeruginosa* increased at 25 °C and 30 °C, with significantly higher GPx activities observed in treatment groups fed high concentrations of toxic *M. aeruginosa* compared to those fed *C. vulgaris* at elevated temperatures and at each sampling time point (*p* < 0.05). The results demonstrated a positive correlation between toxic *M. aeruginosa* exposure and elevated SOD and GPx activities at high temperatures (30 °C), indicating that the SOD and GPx enzymes play a crucial role in antioxidant defense systems and may aid *C. fluminea* in effectively defending against MCs under thermal stress. Additionally, GR activity displayed a significant decrease in treated clams fed toxic *M. aeruginosa* compared to those fed *C. vulgaris* at 25 °C and 30 °C (*p* < 0.05), while this significant inhibition of GR did not appear when exposed to toxic *M. aeruginosa* at 20 °C (Figure 2). Results of the three-way ANOVA demonstrated that toxic *M. aeruginosa* and temperature have significant effects on the activities of SOD, CAT, GPX and GR (*p* < 0.05) (Table 1). Significant interactive effects between toxic *M. aeruginosa* and temperature were also observed on these tested parameters (*p* < 0.05) (Table 1). This is in accordance with the findings of Kim et al. [32,36], who showed that microcystin exposure induces the enzymatic activities of antioxidant defense systems including GST, CAT, SOD, GPx and GR in the bivalves *Crassostrea gigas* and *Mytilus edulis*. A previous study in *C. fluminea* also demonstrated that SOD activity was elevated by toxic *M. aeruginosa* [25]. Our results also agree well with the observations of Liu et al. [9], who found that both toxic *M. aeruginosa* and thermal stress resulted in the enhancement of the enzymatic activities of SOD, CAT and GPx in *H. cumingii*. In *Dreissena polymorpha*, elevated GST and SOD activity was observed after exposure to MC, whereas GST, SOD and CAT activity were inhibited or barely affected in *Unio tumidus* [33]. Different trends in the SOD, CAT, GPx and GR activities observed in various bivalve species could be attributed to interspecies differences as well as the significant influence of thermal stress on basal metabolic levels in bivalves [37].

#### 3.1.3. Assessment of ROS Levels and Lipid Oxidation

**Figure 3:**
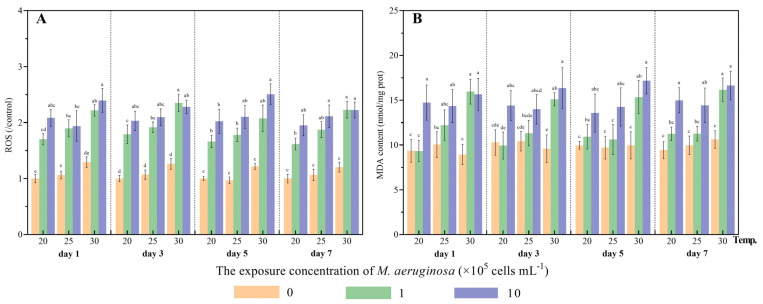
The reactive oxygen species (ROS) (**A**) and malondialdehyde (MDA) (**B**) content of *C*. *fluminea* exposed to nine combinations of toxic *M. aeruginosa* (0, 1 and 10 × 10^5^ cells/mL) and temperatures (20, 25 and 30 °C) at days 1, 3, 5 and 7. Different letters indicate significant differences among treatment groups at each sampling time (Tukey HSD test, *p* < 0.05). Error bars indicate means ± SD (*n* = 3).

The MDA level serves as a significant biomarker of lipid peroxidation, providing information on the degree of cellular oxidative damage caused by excessive ROS [9]. In this study, ROS production increased after toxic *M. aeruginosa* exposure, and a significant increase in ROS was observed in treated clams fed toxic *M. aeruginosa* compared to those fed *C. vulgaris* at the tested temperatures and at each sampling time point (*p* < 0.05) (Figure 3). Comparably, a significant enhancement of MDA content was observed in treated clams fed high concentrations of toxic *M. aeruginosa* compared to those fed *C. vulgaris* (Figure 3). And the MDA content only significantly increased in treated clams fed medium concentrations of toxic *M. aeruginosa* compared to those fed *C. vulgaris* at 30 °C (*p* < 0.05), while no significant increase was observed when the clams were exposed to low temperatures (20 °C) (Figure 3). These results indicated that thermal stress exacerbated oxidative damage in treated clams from the treatment groups receiving a low concentration of toxic *M. aeruginosa*. The three-way ANOVA results showed that toxic *M. aeruginosa* and temperature have significant effects on ROS and MDA (*p* < 0.05) (Table 1). Additionally, significant interactive effects between toxic *M. aeruginosa* and temperature were also observed in ROS and MDA (*p* < 0.05) (Table 1). Similarly, significantly elevated ROS and MDA content was observed in other bivalves, including *H. cumingii*, *C. gigas* and *M. edulis*, after toxic *M. aeruginosa* or MC exposure [9,32].

#### 3.1.4. Integrated Analysis of Biochemical Parameters

**Figure 4:**
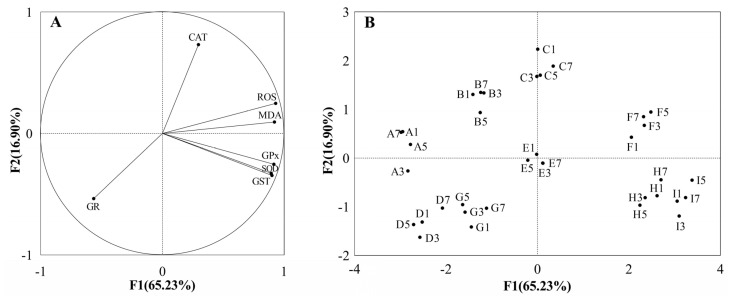
Biplot of principal component analysis (PCA) integrating all tested biochemical parameter variables (including GST, SOD, CAT, GPx and GR activity, ROS level and MDA content) and four time points (days: 1, 3, 5 and 7) at nine different treatments (A: 0 cells/mL *M. aeruginosa* × 20 °C; B: 1 × 10^5^ cells/mL *M. aeruginosa* × 20 °C; C: 10 × 10^5^ cells/mL *M. aeruginosa* × 20 °C; D: 0 cells/mL *M. aeruginosa* × 25 °C; E: 1 × 10^5^ cells/mL *M. aeruginosa* × 25 °C; F: 10 × 10^5^ cells/mL *M. aeruginosa* × 25 °C; G: 0 cells/mL *M. aeruginosa* × 30 °C; H: 1 × 10^5^ cells/mL *M. aeruginosa* × 30 °C; I: 10 × 10^5^ cells/mL *M. aeruginosa* × 30 °C). Both the loadings of the variables (**A**) and the scores of the experimental conditions (**B**) are displayed.

The PCA approach is a factor analysis that achieves a significant reduction in the dimensionality of the original data set, revealing a meaningful association among the variables of interest [38]. In this study, the PCA of the biochemical parameters showed that 82.13% of the total variance was accounted for by the first two principal components (Figure 4A). PC1 accounted for 65.23% of the overall variation, with GR exhibiting the only negative contribution. PC2 was responsible for 16.90% of the total variance and was driven by CAT, ROS and MDA. The PCA results demonstrated that temperature played an important role when toxic *M. aeruginosa* was removed, as evidenced by the pronounced clustering of the three temperature treatments without toxic *M. aeruginosa* exposure (Figure 4B). However, when accompanied by toxic *M. aeruginosa* exposure, the clustering effects resulting from temperature show a degree of attenuation, indicating the apparent interaction between toxic *M. aeruginosa* and thermal stress.

**Figure 5:**
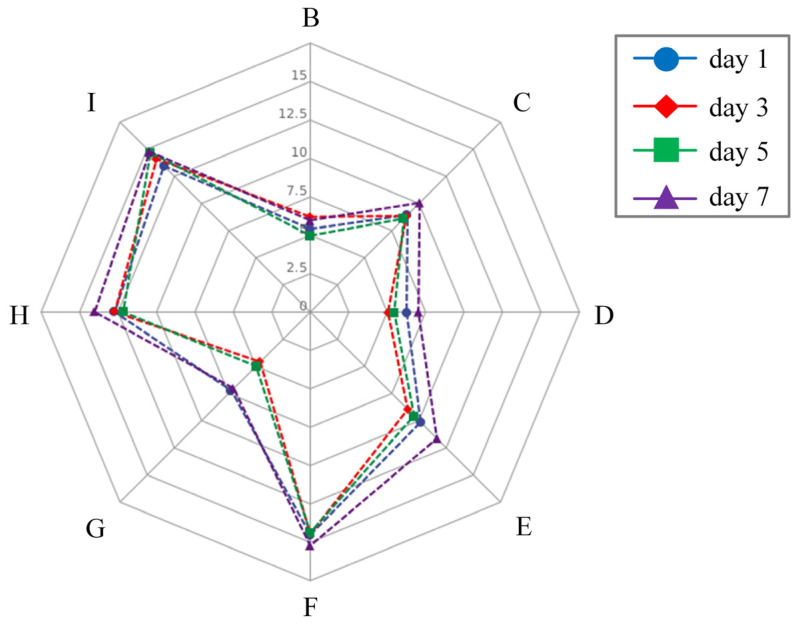
The integrated biomarker response (IBR) values, in the form of a star plot extracted, for different treatment groups (B: 1 × 10^5^ cells/mL *M. aeruginosa* × 20 °C; C: 10 × 10^5^ cells/mL *M. aeruginosa* × 20 °C; D: 0 cells/mL *M. aeruginosa* × 25 °C; E: 1 × 10^5^ cells/mL *M. aeruginosa* × 25 °C; F: 10 × 10^5^ cells/mL *M. aeruginosa* × 25 °C; G: 0 cells/mL *M. aeruginosa* × 30 °C; H: 1 × 10^5^ cells/mL *M. aeruginosa* × 30 °C; I: 10 × 10^5^ cells/mL *M. aeruginosa* × 30 °C) at days 1, 3, 5 and 7. The IBR values were calculated based on all tested biochemical parameters, including GST, SOD, CAT, GPx and GR activity, ROS level and MDA content.

The IBR index is a commonly used quantitative measure in toxicological studies that effectively evaluates individual biomarker responses [39]. It serves as a comprehensive indicator of the health state of aquatic organisms exposed to environmental stressors and toxic substances, integrating all relevant responses [40]. In this study, the IBR values of the treatment groups consistently followed comparable patterns at various time intervals, showing a consistent increase as the concentrations of toxic *M. aeruginosa* increased at the same temperatures (Figure 5). Our observations were consistent with the results of Li et al. [41], who reported that the elevated IBR values integrated with biochemical parameters in the clam *Ruditapes philippinarum* and scallop *Chlamys farreri* were significantly correlated to the chemical contaminants’ concentrations. Similarly, a previous study also found that the IBR values in *R*. *philippinarum* increased after exposure to heavy metal contaminations [42]. In the present study, the results proved that toxic *M. aeruginosa* exposure has an apparent influence on the oxidative stress responses of *C. fluminea* at each temperature.

### 3.2. Molecular Processes Revealed via Transcriptome Analysis

**Figure 6:**
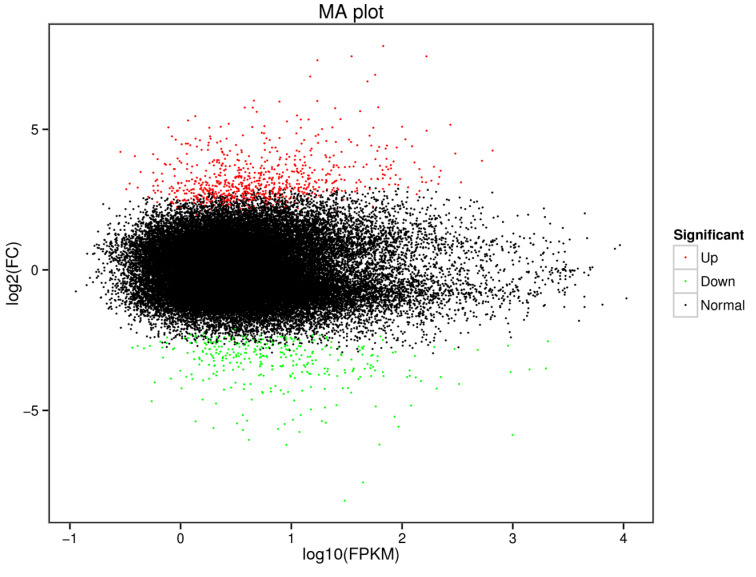
Volcano plot of differentially expressed genes (DEGs) in the hepatopancreas of *C. fluminea* fed with toxic *M. aeruginosa* at 30 °C compared with the control (fed with *C. vulgaris* at 20 °C) at 7 days post-treatment. Red dots represent up-regulated genes and green dots represent down-regulated genes.

To investigate the molecular processes behind the tolerance of *C. fluminea* to MCs under thermal stress, the RNA-Seq approach was employed to examine transcriptome alterations in *C. fluminea* co-exposed to a high concentration of *M. aeruginosa* (10 × 10^5^ cells/mL) and high temperatures (30 °C). After 7 days of co-exposure to *M. aeruginosa* and thermal stress, a total of 1105 DEGs, including 761 significantly up-regulated genes and 344 significantly down-regulated genes, were observed in the treatment group compared to the control group (fed *C. vulgaris* at 20 °C) (Figure 6). Subsequently, the DEGs were subjected to eggNOG and GO annotations, as well as KEGG pathway analysis.

**Figure 7:**
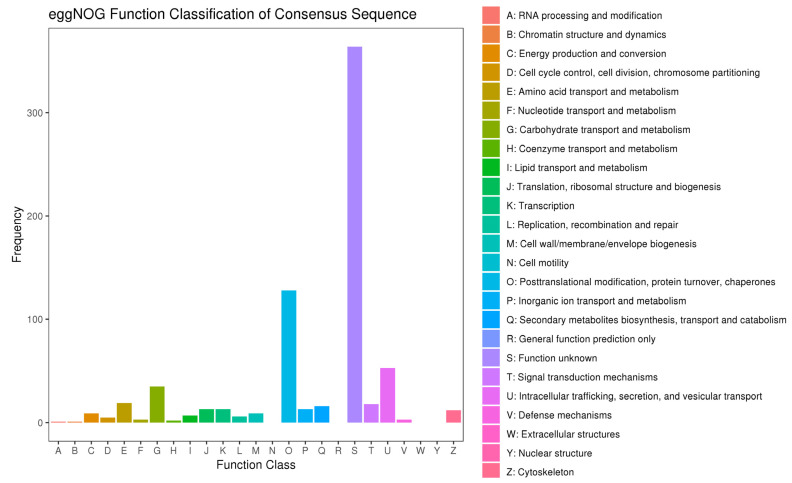
Classification of the differentially expressed genes (DEGs) annotated in eggNOG. The X-axis represents the names of the annotated groups, and the Y-axis corresponds to the percentage of the number of DEGs in the group accounting for the total number of annotated DEGs.

**Figure 8:**
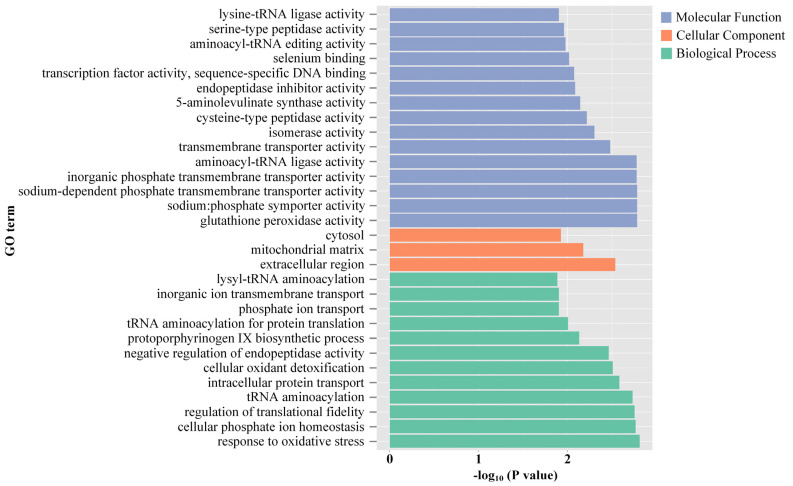
Gene Ontology (GO) categories and the pattern of the differentially expressed genes (DEGs). The distribution of the GO categories was split into three categories: molecular functions (MF), cellular component (CC) and biological process (BP). A lower *p* value represents more significant enrichment.

eggNOG is a publicly accessible database for the fast functional annotation and orthology prediction of custom genomics or metagenomics data sets [43]. This study involved the annotation of 717 DEGs into 21 eggNOG terms. Except for group of function unknown, function class of posttranslational modification, and protein turnover, chaperones have the most DEGs (17.85% of annotated DEGs), followed by intracellular trafficking, secretion, and vesicular transport (7.39%), and carbohydrate transport and metabolism (4.88%) (Figure 7). The top 30 GO terms with the most significant DEGs belong to three domains: molecular function (MF) included 15 subcategories, cellular component (CC) included 3 subcategories, and biological process (BP) included 12 subcategories (Figure 8). In MF, glutathione peroxidase activity, sodium: phosphate symporter activity and sodium-dependent phosphate transmembrane transporter activity constituted the top three clusters. In CC, the top three GO clusters were extracellular region, mitochondrial matrix and cytosol. The three most significant GO groups in BP were responses to oxidative stress, cellular phosphate ion homeostasis and the regulation of translational fidelity. The results revealed by DEG annotations confirmed that a substantial portion of DEGs exhibited associations with responses to oxidative stress and transporter activity which agree well with enzymatic observations in the activation of biotransformation and antioxidant defense systems, suggesting that *C. fluminea* could initiate integrated metabolic processes in response to oxidative stress caused by microcystins and thermal stress.

**Figure 9:**
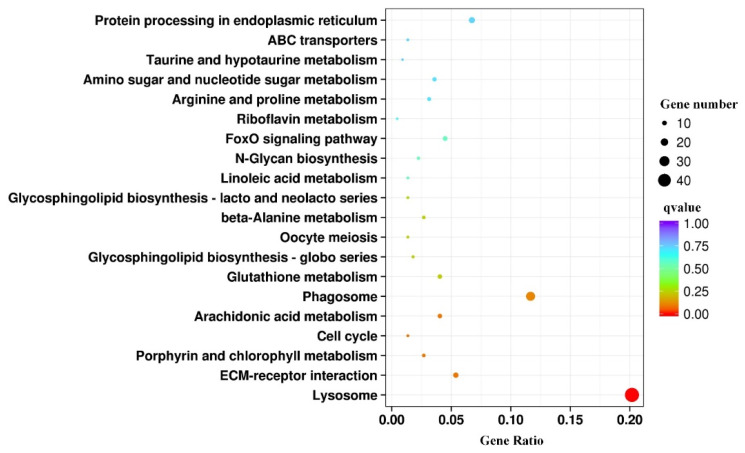
Kyoto Encyclopaedia of Genes and Genomes (KEGG) pathway analysis of the differentially expressed genes (DEGs). Coloring represents the q value. A lower *q* value represents a more significant enrichment. The point size is proportional to the number of DEGs.

The analysis of KEGG functional classifications indicated that the lysosome pathway was significantly enriched after treatment with toxic *M. aeruginosa* and thermal stress (FDR < 0.05) (Figure 9). The three most abundant KEGG pathways were lysosome, phagosome and protein processing in the endoplasmic reticulum, which were closely related to the inflammatory response (Figure 9). Furthermore, other abundant KEGG pathways were involved in immune functions, including the ECM–receptor interaction and FoxO signaling pathway, and metabolism functions, including arachidonic acid metabolism and glutathione metabolism (Figure 9). Arachidonic acid, known as the precursor to prostaglandins, has a wide range of physiological functions, including acting as a pro-inflammatory mediator and promoting the oxidation of exogenous compounds via co-oxidation [44]. The activation of the pathways related to inflammatory and immune responses could be attributed to the oxidative stress induced by high temperatures and MCs. Additionally, the oxidation and co-oxidation occurring intracellularly play a significant role in the metabolic transformation of cyanotoxins into more polar molecules [45]. Subsequently, these polar compounds were subjected to glutathione metabolism-mediated conjugation reactions and eventually discharged [46]. Therefore, it is reasonable to assume that the metabolic processes of co-oxidation (phase I reaction) and conjugation (phase II reaction) in combination contribute to the biodetoxification of MCs in *C. fluminea* under thermal stress.

## 4. Conclusions

This study provides the first comprehensive analysis of the oxidative stress responses and molecular mechanisms in *C. fluminea* under co-exposure to toxic *M*. *aeruginosa* and thermal stress. The results indicated that the antioxidant enzyme activity, ROS levels and MDA content in *C. fluminea* were significantly affected by the interaction between toxic *M. aeruginosa* and *temperature*. The elevated SOD and GPx activities helped *C. fluminea* defend effectively against MCs under thermal stress. Additionally, differentially expressed gene analysis indicated that these responses involved oxidative stress, transporter activity, inflammatory and immune functions and metabolic processes. These findings improve our understanding of the mechanisms of physiological adaptation in bivalves when exposed to both toxic cyanobacteria and thermal stress. It also enables the evaluation of the viability of using bivalves as a bioremediation tool to manage CYBs in eutrophic waters.

## Figures and Tables

**Table 1:** Summary of three-way ANOVA results for the effects of temperature (Temp), toxic *M. aeruginosa* (M) and sampling time points (Time) on biochemical parameters (glutathione S−transferase, GST; superoxide dismutase, SOD; catalase, CAT; glutathione peroxidase, GPx; glutathione reductase, GR; reactive oxygen species, ROS; malondialdehyde, MDA) in *C*. *fluminea* as a function of time. The thermal treatments were 20, 25 and 30 °C The concentrations of toxic *M. aeruginosa* were 0, 1 and 10 × 10^5^ cells/mL. The sampling time points were days 1, 3, 5 and 7.

Factor	Degrees of Freedom	Mean Square	*F*	*p*
GST				
Temp	2	4416.781	133.277	<0.001
Time	3	12.904	0.389	0.761
M	2	1910.061	57.636	<0.001
Temp × Time	6	11.485	0.347	0.91
Temp × M	4	165.007	4.979	0.001
Time × M	6	9.835	0.297	0.937
Temp × Time × M	12	7.466	0.225	0.997
SOD				
Temp	2	8814.217	330.075	<0.001
Time	3	13.086	0.49	0.69
M	2	4884.743	182.924	<0.001
Temp × Time	6	8.155	0.305	0.932
Temp × M	4	498.276	18.659	<0.001
Time × M	6	11.935	0.447	0.845
Temp × Time × M	12	4.756	0.178	0.999
CAT				
Temp	2	629.899	73.301	<0.001
Time	3	12.074	1.405	0.248
M	2	781.737	90.97	<0.001
Temp × Time	6	2.91	0.339	0.914
Temp × M	4	130.706	15.21	<0.001
Time × M	6	4.371	0.509	0.8
Temp × Time × M	12	1.838	0.214	0.997
GPx				
Temp	2	185.395	84.353	<0.001
Time	3	0.162	0.074	0.974
M	2	165.223	75.176	<0.001
Temp × Time	6	3.211	1.461	0.204
Temp × M	4	22.769	10.36	<0.001
Time × M	6	0.794	0.361	0.901
Temp × Time × M	12	2.008	0.914	0.538
GR				
Temp	2	87.409	44.326	<0.001
Time	3	2.804	1.422	0.243
M	2	93.35	47.339	<0.001
Temp × Time	6	0.828	0.42	0.863
Temp × M	4	16.357	8.295	<0.001
Time × M	6	1.441	0.731	0.626
Temp × Time × M	12	0.922	0.468	0.927
ROS				
Temp	2	1.325	64.356	<0.001
Time	3	0.02	0.949	0.421
M	2	11.049	536.777	<0.001
Temp × Time	6	0.01	0.473	0.826
Temp × M	4	0.064	3.112	0.02
Time × M	6	0.029	1.391	0.23
Temp × Time × M	12	0.015	0.715	0.732
MDA				
Temp	2	62.491	28.094	<0.001
Time	3	1.125	0.506	0.68
M	2	242.191	108.883	<0.001
Temp × Time	6	1.093	0.491	0.813
Temp × M	4	25.154	11.309	<0.001
Time × M	6	0.496	0.223	0.968
Temp × Time × M	12	1.221	0.549	0.875

## Data Availability

All of the data are included in the article.

## Supplementary Materials

The following supporting information can be downloaded at https://www.mdpi.com/article/10.3390/antiox12111901/s1, Figure S1: heatmap matrix and chordal graph showing pair correlation coefficients (Pearson correlation) across biochemical parameters.

## Abbreviations

Cyanobacterial blooms (CYBs); microcystins (MCs); reactive oxygen species (ROS); superoxide dismutase (SOD); catalase (CAT); glutathione peroxidase (GPx); glutathione reductase (GR); RNA sequencing (RNA-seq); glutathione S−transferase (GST); malondialdehyde (MDA); principal component analysis (PCA); integrated biomarker response (IBR); fold change (FC); false discovery rate (FDR); differentially expressed genes (DEGs); evolutionary genealogy of genes: Non-supervised Orthologous Groups (eggNOG); Gene Ontology (GO); Kyoto Encyclopaedia of Genes and Genomes (KEGG); analysis of variance (ANOVA); glutathione (GSH); molecular function (MF); cellular component (CC); biological process (BP).

## References

[1] Huisman J., Codd G.A., Paerl H.W., Ibelings B.W., Verspagen J.M., Visser P.M. (2018). Cyanobacterial blooms. Nat. Rev. Microbiol..

[2] Wei H., Jia Y., Wang Z. (2022). Microcystin pollution in lakes and reservoirs: A nationwide meta-analysis and assessment in China. Environ. Pollut..

[3] Shi L., Du X., Liu H., Chen X., Ma Y., Wang R., Tian Z., Zhang S., Guo H., Zhang H. (2021). Update on the adverse effects of microcystins on the liver. Environ. Res..

[4] Díez-Quijada L., Prieto A.I., Guzmán-Guillén R., Jos A., Cameán A.M. (2019). Occurrence and toxicity of microcystin congeners other than MC-LR and MC-RR: A review. Food Chem. Toxicol..

[5] Gobler C.J., Doherty O.M., Hattenrath-Lehmann T.K., Griffith A.W., Kang Y., Litaker R.W. (2017). Ocean warming since 1982 has expanded the niche of toxic algal blooms in the North Atlantic and North Pacific oceans. Proc. Natl. Acad. Sci. USA.

[6] Ho J.C., Michalak A.M., Pahlevan N. (2019). Widespread global increase in intense lake phytoplankton blooms since the 1980s. Nature.

[7] Gobler C.J. (2020). Climate change and harmful algal blooms: Insights and perspective. Harmful Algae.

[8] Griffith A.W., Gobler C.J. (2020). Harmful algal blooms: A climate change co-stressor in marine and freshwater ecosystems. Harmful Algae.

[9] Liu Y., Yang M., Zheng L., Nguyen H., Ni L., Song S., Sui Y. (2020). Antioxidant responses of triangle sail mussel *Hyriopsis cumingii* exposed to toxic *Microcystis aeruginosa* and thermal stress. Sci. Total Environ..

[10] Zhang X., Ji W., Zhang H., Zhang W., Xie P. (2011). Studies on the toxic effects of microcystin-LR on the zebrafish (*Danio rerio*) under different temperatures. J. Appl. Toxicol..

[11] Ji W., Liang H., Zhou W., Zhang X. (2013). Apoptotic responses of zebrafish (*Danio rerio*) after exposure with microcystin-LR under different ambient temperatures. J. Appl. Toxicol..

[12] Kim J., Seo J.K., Yoon H., Kim P.J., Choi K. (2014). Combined effects of the cyanobacterial toxin microcystin-LR and environmental factors on life-history traits of indigenous cladoceran *Moina macrocopa*. Environ. Toxicol. Chem..

[13] Liang Y., Gao T., Shao L., Min Y., Yang J. (2020). Effects of microcystin-LR and nitrite on the lifespan, reproduction, and heat shock responses of rotifer *Brachionus calyciflorus* at different temperatures. Aquat. Sci..

[14] Higgins S.N., Zanden M.V. (2010). What a difference a species makes: A meta-analysis of dreissenid mussel impacts on freshwater ecosystems. Ecol. Monogr..

[15] Lance E., Lepoutre A., Savar V., Robert E., Bormans M., Amzil Z. (2021). In situ use of bivalves and passive samplers to reveal water contamination by microcystins along a freshwater-marine continuum in France. Water Res..

[16] Haag W.R., Culp J., Drayer A.N., McGregor M.A., White D.E., Price S.J. (2021). Abundance of an invasive bivalve, *Corbicula fluminea*, is negatively related to growth of freshwater mussels in the wild. Freshw. Biol..

[17] Pham T.L., Shimizu K., Dao T.S., Motoo U. (2017). First report on free and covalently bound microcystins in fish and bivalves from Vietnam: Assessment of risks to humans. Environ. Toxicol. Chem..

[18] Burket S.R., White M., Ramirez A.J., Stanley J.K., Banks K.E., Waller W.T., Chambliss C.K., Brooks B.W. (2019). *Corbicula fluminea* rapidly accumulate pharmaceuticals from an effluent dependent urban stream. Chemosphere.

[19] Shan Y., Yan S., Hong X., Zha J., Qin J. (2020). Effect of imidacloprid on the behavior, antioxidant system, multixenobiotic resistance, and histopathology of Asian freshwater clams (*Corbicula fluminea*). Aquat. Toxicol..

[20] Yan S., Wang Q., Yang L., Zha J. (2020). Comparison of the toxicity effects of tris (1, 3-dichloro-2-propyl) phosphate (TDCIPP) with tributyl phosphate (TNBP) reveals the mechanism of the apoptosis pathway in Asian freshwater clams (*Corbicula fluminea*). Environ. Sci. Technol..

[21] Shen R., Gu X., Chen H., Mao Z., Zeng Q., Jeppesen E. (2020). Combining bivalve (*Corbicula fluminea*) and filter-feeding fish (*Aristichthys nobilis*) enhances the bioremediation effect of algae: An outdoor mesocosm study. Sci. Total Environ..

[22] Zhang Y., Shen R., Gu X., Li K., Chen H., He H., Mao Z., Johnson R.K. (2023). Simultaneous increases of filter-feeding fish and bivalves are key for controlling cyanobacterial blooms in a shallow eutrophic lake. Water Res..

[23] Castañeda R.A., Cvetanovska E., Hamelin K.M., Simard M.A., Ricciardi A. (2018). Distribution, abundance and condition of an invasive bivalve (*Corbicula fluminea*) along an artificial thermal gradient in the St. Lawrence River. Aquat. Invasions.

[24] Penk M.R., Williams M.A. (2019). Thermal effluents from power plants boost performance of the invasive clam *Corbicula fluminea* in Ireland’s largest river. Sci. Total Environ..

[25] Zhang J., Yu M., Gao Y., Zhang M., Dong J., Li M., Li X. (2023). Feeding behavior, microcystin accumulation, biochemical response, and ultramicrostructure changes in edible freshwater bivalve *Corbicula fluminea* exposed to *Microcystis aeruginosa*. Environ. Sci. Pollut. Res..

[26] Amado L.L., Monserrat J.M. (2010). Oxidative stress generation by microcystins in aquatic animals: Why and how. Environ. Int..

[27] Wang Z., Li G., Wu Q., Liu C., Shen J., Yan W. (2019). Microcystin-LR exposure induced nephrotoxicity by triggering apoptosis in female zebrafish. Chemosphere.

[28] Zhang Y., Li Z., Kholodkevich S., Sharov A., Feng Y., Ren N., Sun K. (2020). Microcystin-LR-induced changes of hepatopancreatic transcriptome, intestinal microbiota, and histopathology of freshwater crayfish (*Procambarus clarkii*). Sci. Total Environ..

[29] Sanchez W., Burgeot T., Porcher J.M. (2013). A novel “Integrated Biomarker Response” calculation based on reference deviation concept. Environ. Sci. Pollut. Res..

[30] Anders S., Huber W. (2010). Differential expression analysis for sequence count data. Genome Biol..

[31] Li Z., Chang X., Hu M., Fang J.K.H., Sokolova I.M., Huang W., Xu E.G., Wang Y. (2022). Is microplastic an oxidative stressor? Evidence from a meta-analysis on bivalves. J. Hazard. Mater..

[32] Kim Y.D., Kim W.J., Shin Y.K., Lee D.H., Kim Y.J., Kim J.K., Rhee J.S. (2017). Microcystin-LR bioconcentration induces antioxidant responses in the digestive gland of two marine bivalves *Crassostrea gigas* and *Mytilus edulis*. Aquat. Toxicol..

[33] Burmester V., Nimptsch J., Wiegand C. (2012). Adaptation of freshwater mussels to cyanobacterial toxins: Response of the biotransformation and antioxidant enzymes. Ecotoxicol. Environ. Saf..

[34] Oyaneder-Terrazas J., Figueroa D., Araneda O.F., García C. (2022). Saxitoxin group toxins accumulation induces antioxidant responses in tissues of *Mytilus chilensis*, *Ameghinomya antiqua*, and *Concholepas concholepas* during a bloom of *Alexandrium pacificum*. Antioxidants.

[35] Martins N.D., Yunes J.S., Mckenzie D.J., Rantin F.T., Kalinin A.L., Monteiro D.A. (2019). Microcystin-LR exposure causes cardiorespiratory impairments and tissue oxidative damage in trahira, *Hoplias malabaricus*. Ecotoxicol. Environ. Saf..

[36] Kim B.M., Haque M.N., Lee D.H., Nam S.E., Rhee J.S. (2018). Comparative toxicokinetics and antioxidant response in the microcystin-LR-exposed gill of two marine bivalves, *Crassostrea gigas* and *Mytilus edulis*. J. Shellfish Res..

[37] Sokolova I.M. (2013). Energy-limited tolerance to stress as a conceptual framework to integrate the effects of multiple stressors. Integr. Comp. Biol..

[38] Wang J., Fu Z., Qiao H., Liu F. (2019). Assessment of eutrophication and water quality in the estuarine area of Lake Wuli, Lake Taihu, China. Sci. Total Environ..

[39] Cravo A., Pereira C., Gomes T., Cardoso C., Serafim A., Almeida C., Rocha T., Lopes B., Company R., Medeiros A. (2012). A multibiomarker approach in the clam *Ruditapes decussatus* to assess the impact of pollution in the Ria Formosa lagoon, South Coast of Portugal. Mar. Environ. Res..

[40] Li Z., Feng C., Wu Y., Guo X. (2020). Impacts of nanoplastics on bivalve: Fluorescence tracing of organ accumulation, oxidative stress and damage. J. Hazard. Mater..

[41] Li Z., Pan L., Guo R., Cao Y., Sun J.A. (2020). verification of correlation between chemical monitoring and multi-biomarker approach using clam *Ruditapes philippinarum* and scallop *Chlamys farreri* to assess the impact of pollution in Shandong coastal area of China. Mar. Pollut. Bull..

[42] Aouini F., Trombini C., Sendra M., Blasco J. (2019). Biochemical response of the clam *Ruditapes philippinarum* to silver (AgD and AgNPs) exposure and application of an integrated biomarker response approach. Mar. Environ. Res..

[43] Huerta-Cepas J., Szklarczyk D., Heller D., Hernández-Plaza A., Forslund S.K., Cook H., Mende D.R., Letunic I., Rattei T., Jensen L.J. (2019). eggNOG 5.0: A hierarchical, functionally and phylogenetically annotated orthology resource based on 5090 organisms and 2502 viruses. Nucleic Acids Res..

[44] Duan Y., Xiong D., Wang Y., Li H., Dong H., Zhang J. (2021). Toxic effects of ammonia and thermal stress on the intestinal microbiota and transcriptomic and metabolomic responses of *Litopenaeus vannamei*. Sci. Total Environ..

[45] Banerjee S., Maity S., Guchhait R., Chatterjee A., Biswas C., Adhikari M., Pramanick K. (2021). Toxic effects of cyanotoxins in teleost fish: A comprehensive review. Aquat. Toxicol..

[46] Krausfeldt L.E., Steffen M.M., McKay R.M., Bullerjahn G.S., Boyer G.L., Wilhelm S.W. (2019). Insight into the molecular mechanisms for microcystin biodegradation in Lake Erie and Lake Taihu. Front. Microbiol..

