# Supplementary material for: The Combined Effects of Toxic Microcystis aeruginosa and Thermal Stress on the Edible Clam (Corbicula fluminea): Insights into Oxidative Stress Responses and Molecular Networks
Source: Antioxidants (Basel). 2023 Oct 24;12(11):1901. doi: 10.3390/antiox12111901 (PMC10669901; doi:10.3390/antiox12111901)

Figure S1. Heatmap matrix (A) and chordal graph (B) showing pair correlation coefficients (Pearson correlation) across biochemical parameters (glutathione S-transferase, GST; superoxide dismutase, SOD; catalase, CAT; glutathione peroxidase, GPx; glutathione reductase, GR; reactive oxygen species, ROS; malondialdehyde, MDA). Significant differences are shown as * $p < 0.05$, ** $p < 0.01$ and ** $p < 0.001$.

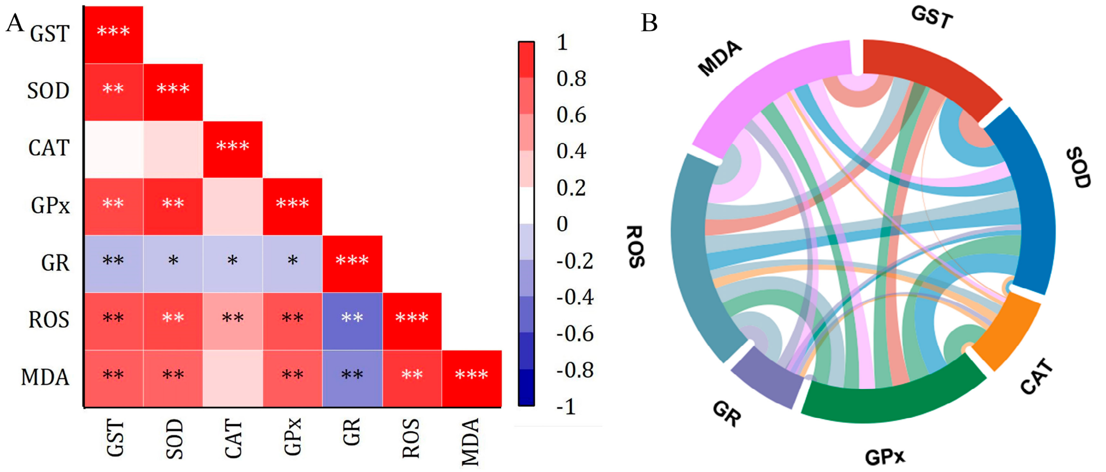

Supplement: Supplementary file 1 [file antioxidants-12-01901-s001.zip › antioxidants-2667429-SI.pdf]
